# ﻿*Sinoseneciotongziensis* (Asteraceae), a new species from north Guizhou, China

**DOI:** 10.3897/phytokeys.252.141719

**Published:** 2025-02-20

**Authors:** Ren-Bo Zhang, Tan Deng, Ying Liu, Da-Jun Xie, Ruo-Xun Wei, Lin He, Quan-Li Dou, Zheng-Min Qian

**Affiliations:** 1 Department of Biology, Zunyi Normal College, Zunyi, CN-563000 Guizhou, China Zunyi Normal College Zunyi China; 2 School of Ecology, Sun Yat-sen University, Shenzhen 518107, China Sun Yat-sen University Shenzhen China; 3 State Key Laboratory of Biocontrol and Guangdong Provincial Key Laboratory of Plant Stress Biology, Sun Yat-sen University, Guangzhou 510275, China Sun Yat-sen University Guangzhou China; 4 Sichuan Academy of Forestry, Chengdu 610081, Sichuan, China Sichuan Academy of Forestry Chengdu China

**Keywords:** Flora of Guizhou, ITS, new taxon, taxonomy

## Abstract

*Sinoseneciotongziensis* R.B.Zhang, Tan Deng & Ying Liu (Asteraceae), a new species from Tongzi County in northern Guizhou, China, is described and illustrated. It closely resembles *S.changii* in the subscapigerous habit, ovate-oblong and pinnately veined leaf lamina, and simple to compound terminal corymbs, yet differs markedly by the texture of leaf lamina (membranous vs. papery), the number of lateral veins (8–10 vs. 10–18), and the indumentum on the stems, leaves, and inflorescences (pubescent with 2–5 mm long, uniseriate, spreading hairs vs. sparsely white arachnoid to densely white tomentose). Phylogenetic analysis indicates that *S.tongziensis* is related to *S.bodinieri*, *S.nanchuanicus*, *S.confervifer*, and S.globigervar.adenophyllus.

## ﻿Introduction

*Sinosenecio* B. Nordenstam (Asteraceae, Senecioneae) is a genus comprising 48 species (Chen et al. 2011; Liu and Yang 2012; Liu et al. 2019; Zou et al. 2020; Chen et al. 2022; Peng et al. 2022; Su et al. 2023a; Su et al. 2023b), characterized by subscapiform or leafy stems, palmately or rarely pinnately veined leaf lamina, solitary to numerous capitula, and often ecalyculate involucres (Peng et al. 2022). Multiple lines of evidence indicate that *Sinosenecio* as currently defined is polyphyletic (Wang et al. 2009; Liu 2010; Liu and Yang 2011a, b; Ren et al. 2017). Species with a base chromosome number of *x* = 30 and strictly polarized endothecial cell wall thickenings are phylogenetically close to the tussilaginoid genera, while the remaining species with *x* = 24 (rarely 13) and both polarized and radial thickenings are closely related to *Nemosenecio* (Kitamura) B. Nordenstam and *Tephroseris* (Reichenbach) Reichenbach.

During a field expedition in 2020, a previously undocumented *Sinosenecio* species was discovered in Tongzi County, north Guizhou, China. At first glance, it closely resembles *Sinoseneciochangii* (B. Nordenstam) B. Nordenstam, a species with *x* = 24, in the subscapigerous habit, pinnately veined and ovate-oblong leaf lamina, and simple to compound terminal corymbs. Based on these characteristics, we determined that this plant represents a new species, described here as *S.tongziensis* with a report on its floral micromorphological characters. Additionally, we performed phylogenetic analysis using nuclear ribosomal internal transcribed spacer (nrITS) sequence data to explore its phylogenetic affiliation in the genus.

## ﻿Material and methods

### ﻿Morphological comparison

For the description of the new species, living plants and dried specimens were examined and measured. The terminology followed Chen (1999), Chen et al. (2011), and Hickey and King (2021). Type specimens of the new species were deposited in ZY, PE, and SCFI. Morphological data of related species were gathered through field work and by examining high-resolution photographs of herbarium specimens from BNU, CAS, CDBI, CSFI, GZTM, HGAS, HWA, IBK, JIU, JJF, KUN, PE, SCFI, WH, ZY. Additionally, observations of living plants were supplemented using resources from online databases such as National Specimen Information Infrastructure platform (Ministry of Science and Technology of China 2013) and the Plant Photo Bank of China website (Institute of Botany of Chinese Academy of Sciences 2008).

### ﻿Phylogenetic analysis

To test the phylogenetic affiliation of *S.tongziensis*, we assembled an ITS dataset containing 55 accessions representing *S.tongziensis*, 39 species of *Sinosenecio*, four of *Nemosenecio*, six of *Tephroseris*, and an outgroup *Petasitestricholobus* Franch. The nrITS sequences of *S.tongziensis* was newly generated for this study, while the remaining sequences were downloaded from GenBank. GenBank accession numbers are provided in Suppl. material 1.

Leaf material of the new species was collected and dried with silica-gel for DNA extraction. Total DNA was extracted using a modified CTAB procedure (Doyle and Doyle 1987). The nrITS region of *S.tongziensis* was amplified and sequenced using primers ITS1 and ITS4 (Doyle and Doyle 1987) following the procedure described in Peng et al. (2022). Sequences were aligned in MEGA7 (Kumar et al. 2016). The final matrix contained 630 characters. The best-fitting model GTR+G was selected based on Akaike information criterion (AIC) in MrMTgui (Nuin 2004). Bayesian inference (BI) analysis was performed using MrBayes 3.2.6 (Ronquist et al. 2012), with four simultaneous Markov chain Monte Carlo (MCMC) chains run for 2,000,000 generations, sampling one tree every 100 generations. We verified that the average deviation of split frequencies had reached a value below 0.01. The first 25% trees were discarded as burn-in and the remaining trees were used to construct a majority-rule consensus tree with Bayesian posterior probabilities (PP).

### ﻿Taxonomic treatment

#### ﻿ Sinosenecio tongziensis

Taxon classificationPlantaeAsteralesAsteraceae

R.B.Zhang, Tan Deng & Ying Liu sp. nov.

5DF5DC9E-D986-5637-AA20-2B9DD5F58A93

urn:lsid:ipni.org:names:77356936-1

Figs 1 , 2

##### Type.

China • Guizhou Province, Tongzi County, Guancang Town, Xianrenshan Mountain, elev. 1,200–1,300 m, growing on slopes beneath forests in karst areas, 27 April 2020, Chong-Bo Ma ZRB1607 (fl.) (***holotype***: ZY!; ***isotype***: PE!), • 5 May 2024, Ren-Bo Zhang ZRB2661 (fl.) (***paratype***: ZY!, SCFI!), • 25 May 2024, Ren-Bo Zhang ZRB2676 (fr.) (***paratype***: ZY!).

##### Diagnosis.

Resembles *S.changii* in the subscapigerous habit, ovate-oblong and pinnately veined leaf lamina, and simple to compound terminal corymbs, but differs by membranous leaf lamina (vs. papery), 8–10 lateral veins (vs. 10–18), and stems and leaves pubescent with 2–5 mm long, uniseriate, spreading hairs (vs. sparsely white arachnoid to densely white tomentose) (Table 1).

**Figure 1.**
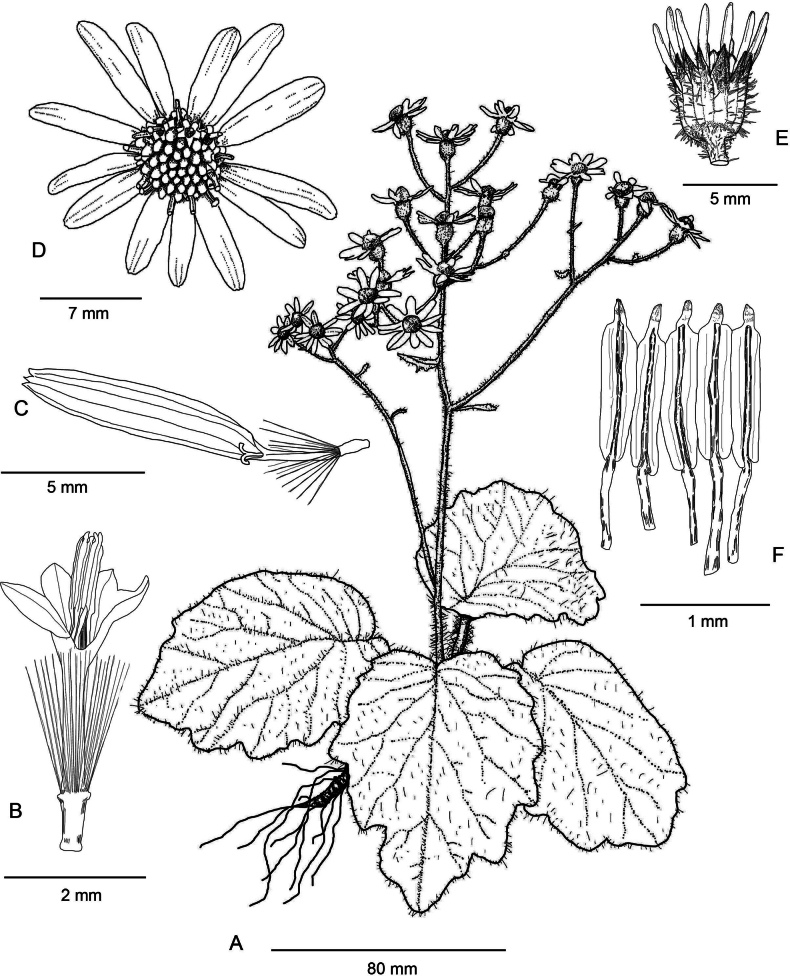
Line drawing of *Sinoseneciotongziensis***A** habit **B** a disk floret **C** a ray floret **D** top view of a capitulum **E** involucre **F** stamens. All from *Chong-Bo Ma ZRB1607* (PE, ZY). Drawn by Tan Deng.

**Table 1.** Comparison of *Sinoseneciotongziensis* and *S.changii*.

Characters	*S.tongziensis*	*S.changii*
Number of cauline leaf	0	usually 1
Indumentum on stems and leaves	Villous with 2–5 mm long, uniseriate, spreading hairs	Sparsely white arachnoid to densely white tomentose
Length of petiole	3–9 cm	2.5–4 cm
Shape of lamina	Ovate, obovate, or ovate-oblong	Ovate-oblong
Size of lamina	4–14 × 3–10 cm	2.5–9 × 2–6 cm
Texture of lamina	Membranous	Papery
Morphology of lamina margin	Long ciliate	Not ciliate
Length of peduncle	1–5 cm	1–4.5 cm
Shape of involucre	Campanulate	Broadly campanulate to hemispheric
Number of phyllaries	13	13
Number of ray florets	10–13	12–18
Achene length	1.6–2.0 mm	ca. 1.5 mm
Flowering	Apr–May	May–Jul
Fruiting	May–Jun	Jun–Aug
Distribution	N Guizhou	S Chongqing, N Guizhou, SE Sichuan

##### Description.

***Herbs***, scapigerous. Fresh ***rhizomes*** 6–16 mm in diam., clad in persistent brown petiole bases. ***Stems*** 1 to 3, erect, scapiform, 15–40 cm tall, ribbed, villous with 2–5 mm long, uniseriate, spreading hairs. ***Leaves*** several, radical, rosulate, densely villous as the stems; petiole 3–9 cm long; blade (broadly) ovate, obovate, or ovate-oblong, 4–14 × 3–10 cm, membranous, villous, densely so along veins, pinnately veined, lateral veins 4–5 pairs, base cordate, margin repand with mucronulate teeth, long ciliate. ***Capitula*** 4–29, arranged in terminal simple to compound corymbs; peduncles 1–5 cm long, slender, (sparsely) pubescent, with 3–20 mm long, linear or linear-spatulate bracts. ***Involucres*** campanulate, 4–7 × 4–7 mm, not calyculate; phyllaries ca. 13, lanceolate, oblanceolate or subelliptic, 4–6 × 1–1.6 mm, herbaceous with membranous margins, abaxially pubescent with uniseriate, spreading hairs, apically acuminate, ciliate at the apex and on upper margin. ***Ray florets*** 10–13; corolla tube 2.5–3.5 mm long, glabrous; ray yellow, oblong, 7–10 × 1.2–2.2 mm, 4-veined, apically 3-denticulate. ***Disk florets*** many; corolla yellow, 4–6 mm long, with 2–3 mm tube and campanulate limb; lobes ovate-lanceolate, ca. 0.8 mm long, apically acute. ***Anthers*** oblong, ca. 1.1 mm long, base obtuse to rounded, appendages lanceolate. ***Styles*** ca. 2.5 mm long in ray florets and 4–4.5 mm long in disk florets, branches recurved, ca. 0.8 mm long. ***Achenes*** cylindric, 1.6–2.0 mm long, inconspicuously ridged, glabrous. ***Pappus*** capillary-form, uniform, white, persistent, 2.4–4.0 mm long.

**Figure 2.**
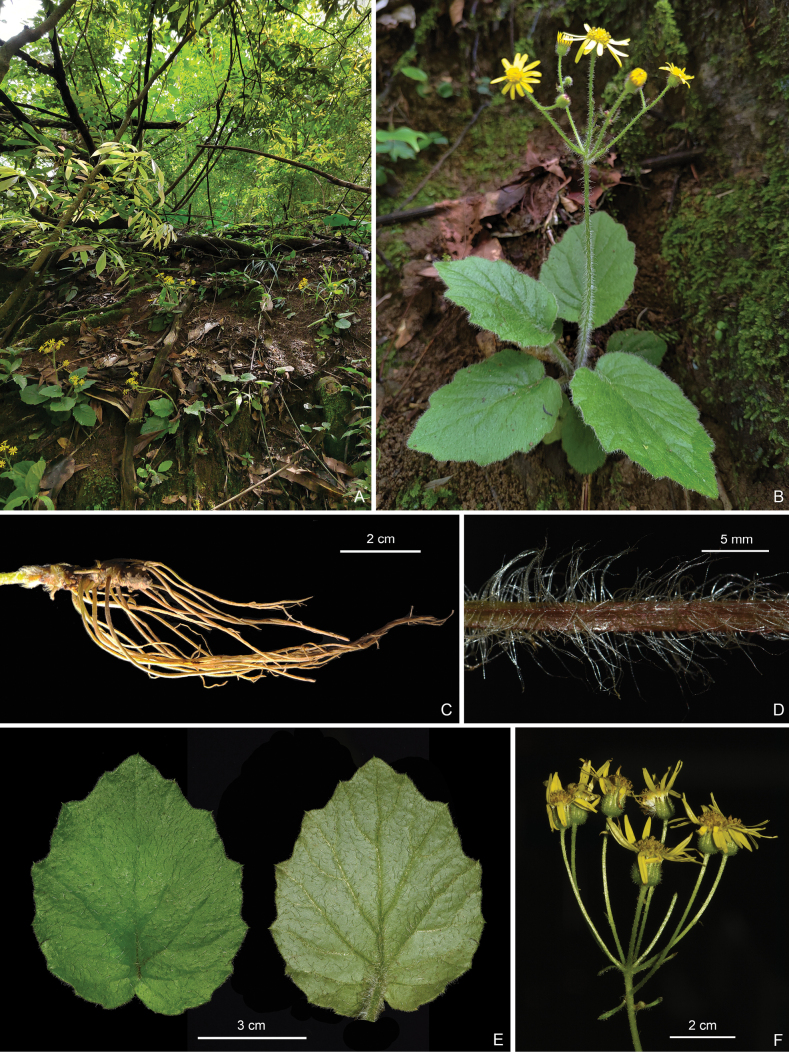
*Sinoseneciotongziensis***A** habitat **B** habit **C** rhizome and fibrous roots **D** close-up of a petiole **E** adaxial (left) and abaxial (right) leaf surface **F** inflorescence (Photographed by Ying Liu and R.B. Zhang).

##### Floral micromorphological characters.

**Figure 3.**
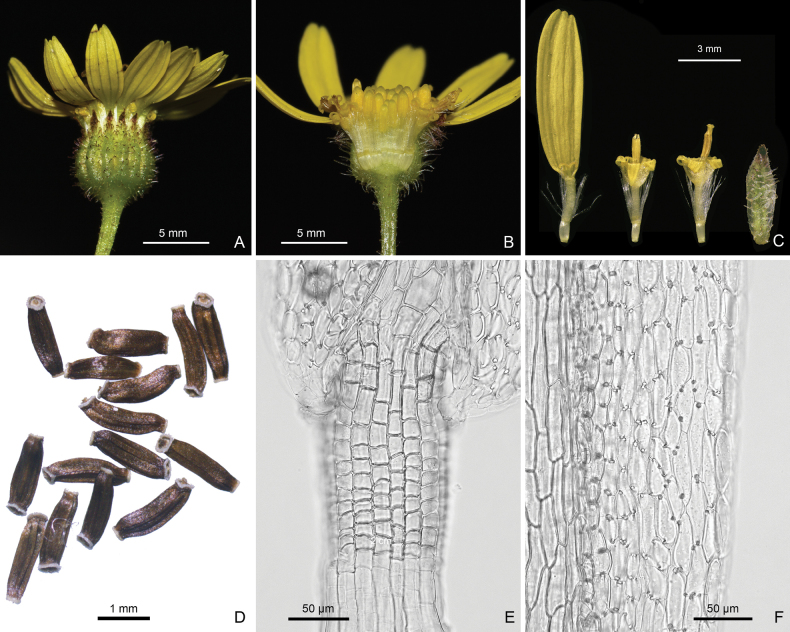
Capitulum, florets, achenes, and floral micromorphological characters of *Sinoseneciotongziensis***A** side view of a capitulum **B** longitudinal section of a capitulum **C** from left to right, a ray floret, two disc florets, and abaxial surface of a phyllary **D** achenes **E** uniformly-sized cells of filament collar **F** polarized and radial anther endothecial cell wall thickenings (Photographed by Ying Liu and R.B. Zhang).

The filament collar of *S.tongziensis* consisted of uniformly sized cells (Fig. 3E), and the anther endothecial cell wall thickenings were polarized and radial (Fig. 3F), lending strong support for the phylogenetic affiliation of *S.tongziensis* in subtribe Tephroseridinae (see below).

##### Phenology.

Flowering from April to May, fruiting from May to June.

##### Etymology.

The specific epithet is derived from the type locality, Tongzi County, Guizhou Province, China.

##### Vernacular name.

The proposed Chinese name is “桐梓蒲儿根”, pronounced as “tóng zǐ pú ér gēn”.

##### Distribution and ecology.

The new species is endemic to Guizhou Province and is currently known only from the type locality, Xianrenshan Mountain, Tongzi County. It grows on slopes beneath the forests in karst areas at elevations of 1,200–1,300 m.

##### Conservation status.

*Sinoseneciotongziensis* is currently recorded only at the type locality. Thousands of individuals are scattered across the mid-slope and at the base of the mountain, with approximately 600 to 700 mature individuals. Given its narrow distribution and relatively low number of mature plants, *S.tongziensis* may be more appropriately categorized as vulnerable (VU) according to the IUCN Red List Categories and Criteria (IUCN Standards and Petitions Committee 2022).

##### Phylogenetic affiliation.

**Figure 4.**
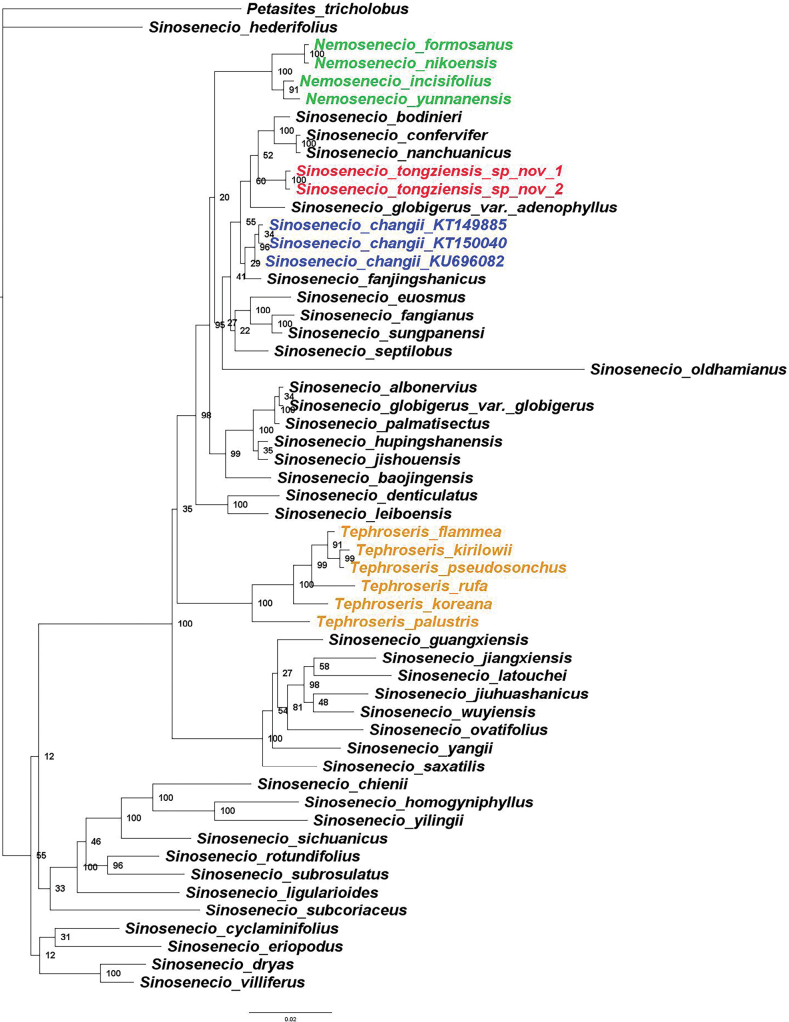
Bayesian phylogenetic tree based on ITS sequence data, showing the phylogenetic position of *Sinoseneciotongziensis*. Numbers at the nodes are Bayesian posterior probabilities. *Nemosenecio* and *Tephroseris* are highlighted in green and yellow, while *S.tongziensis* and *S.changii* are noted in red and blue respectively. GenBank accession numbers were provided for three accessions of *S.changii*.

In the phylogenetic tree, *Nemosenecio* and *Tephroseris* were resolved as monophyletic (Fig. 4). Together with some species of *Sinosenecio*, these genera constituted a well-resolved clade representing subtribe Tephroseridinae (Fig. 4), conforming to previous studies (Wang et al. 2009; Ren et al. 2017; Zou et al. 2020). Despite its close resemblance to *S.changii*, *S.tongziensis* instead formed a weakly supported lineage (PP = 0.6) with *S.bodinieri* (Vaniot) B. Nordenstam, *S.confervifer* (H. Léveillé) Y. Liu & Q. E. Yang, *S.nanchuanicus* Z. Y. Liu, Y. Liu & Q. E. Yang, and S.globigervar.adenophyllus C.Jeffrey & Y.L.Chen (Fig. 4). It can be easily distinguished from these related species by the pinnately veined leaves (vs. palmately).

## Supplementary Material

XML Treatment for Sinosenecio tongziensis

## ﻿Additional information

### Author ORCIDs

Ren-Bo Zhang https://orcid.org/0000-0002-9860-5803

### Data availability

All of the data that support the findings of this study are available in the main text or Supplementary Information.

## Supplementary materials

Supplementary material 1 Supplementary information Data type xlsx https://binary.pensoft.net/file/1256551This dataset is made available under the Open Database License (http://opendatacommons.org/licenses/odbl/1.0/). The Open Database License (ODbL) is a license agreement intended to allow users to freely share, modify, and use this Dataset while maintaining this same freedom for others, provided that the original source and author(s) are credited.Ren-Bo Zhang, Tan Deng, Ying Liu, Da-Jun Xie, Ruo-Xun Wei, Lin He, Quan-Li Dou, Zheng-Min Qian
